# Selective Host Cell Death by *Staphylococcus aureus*: A Strategy for Bacterial Persistence

**DOI:** 10.3389/fimmu.2020.621733

**Published:** 2021-01-21

**Authors:** Dominique Missiakas, Volker Winstel

**Affiliations:** ^1^Howard Taylor Ricketts Laboratory, Department of Microbiology, University of Chicago, Lemont, IL, United States; ^2^Research Group Pathogenesis of Bacterial Infections, TWINCORE, Centre for Experimental and Clinical Infection Research, a joint venture between the Hannover Medical School and the Helmholtz Centre for Infection Research, Hannover, Germany; ^3^Institute of Medical Microbiology and Hospital Epidemiology, Hannover Medical School, Hannover, Germany

**Keywords:** *Staphylococcus aureus*, host cell death, persistence, infection, abscess, blood stream infection

## Abstract

Host cell death programs are fundamental processes that shape cellular homeostasis, embryonic development, and tissue regeneration. Death signaling and downstream host cell responses are not only critical to guide mammalian development, they often act as terminal responses to invading pathogens. Here, we briefly review and contrast how invading pathogens and specifically *Staphylococcus aureus* manipulate apoptotic, necroptotic, and pyroptotic cell death modes to establish infection. Rather than invading host cells, *S. aureus* subverts these cells to produce diffusible molecules that cause death of neighboring hematopoietic cells and thus shapes an immune environment conducive to persistence. The exploitation of cell death pathways by *S. aureus* is yet another virulence strategy that must be juxtaposed to mechanisms of immune evasion, autophagy escape, and tolerance to intracellular killing, and brings us closer to the true portrait of this pathogen for the design of effective therapeutics and intervention strategies.

## Introduction

Human innate immune defenses substantially contribute to microbial clearance during infection (1, 2). Primary defenses encompass mechanisms that include the biosynthesis of antimicrobial peptides on the skin or mucosal surfaces, the recruitment of immune cells to infectious foci, and the activation of the complement system and coagulation cascade (2–4). Programmed cell death modalities represent additional key mechanisms that affect host-microbe interaction and infection control (5). Amongst cell death programs, conventional apoptosis, regulated necrosis (necroptosis), and pyroptosis have thoroughly been described to reveal unique signaling routes for initiation and execution of cell death (6, 7). Signaling and ensuing death modes are governed by the nature of the infection and the pathogenic attributes of the invading microbe. For example, apoptosis is often activated to release intracellular pathogens from infected host cells or tissues (8). In this manner, the host removes a preferred niche for initial replication, and simultaneously exposes the pathogen to extracellular immune cell defenses without causing inflammation. On the contrary, necroptosis and pyroptosis are highly inflammatory and impact immune cell trafficking as well as clinical syndromes and host-mediated clearance of pathogenic microorganisms (5).

**Figure 1:**
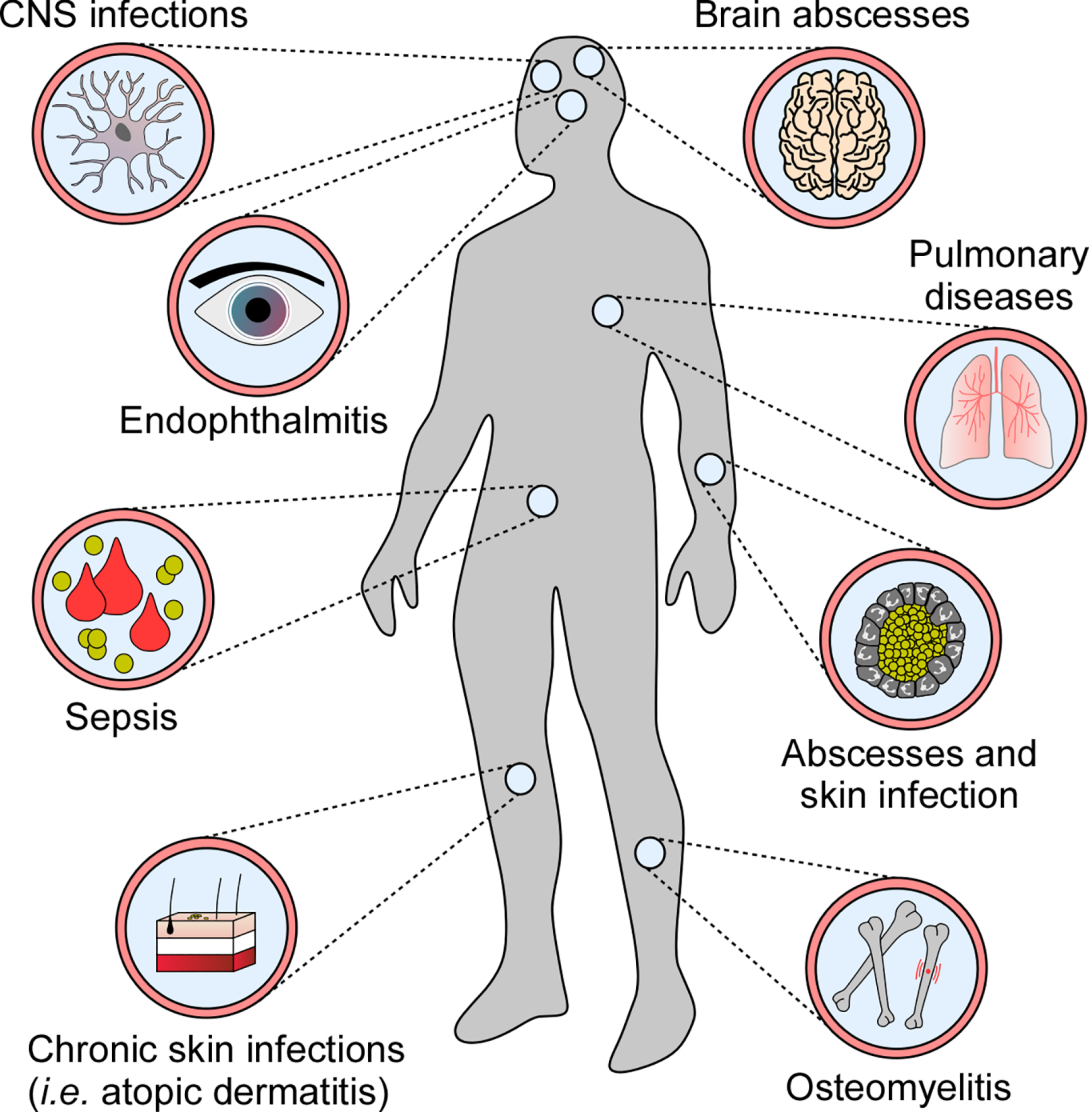
Staphylococcal diseases associated with programmed cell death. *S. aureus* exploits programmed cell death to cause various diseases in human and animal hosts.

Many human pathogens have evolved sophisticated strategies to modulate or subvert host cell death programs during infection (9). Specifically, microbes that infiltrate host cells and replicate intracellularly suppress death signaling pathways to escape extracellular immuno-surveillance (10, 11). In this manner, intracellular bacterial pathogens such as *Mycobacterium tuberculosis* or *Legionella pneumophila* maintain their proliferative niche to cause persistent infections (12–14). Yet, not all pathogens block cell death modalities upon host invasion. Some infectious agents, such as *Staphylococcus aureus*, induce or exploit programmed cell death to establish infection and disseminate in the host. *S. aureus* is the most frequently encountered agent of superficial skin and soft tissue infections and occasionally causes invasive diseases in humans. Once disseminated through blood stream infection, *S. aureus* is able to establish replication foci in almost any organ (**Figure 1**) (15, 16). *S. aureus* deploys an arsenal of virulence factors with potent immunomodulatory or toxigenic properties that modulate programmed cell death in professional and non-professional phagocytes thereby affecting clinical syndromes and various diseases in human or animal hosts (**Figure 1**) (17, 18). Overall, this remarkable microbe has evolved to manipulate all known principal mechanisms of programmed cell death, including apoptosis and pro-inflammatory necroptotic or pyroptotic cell death.

Herein, we summarize various cell death modalities and their impact on the pathogenesis of *S. aureus* infections. We provide an overview of *S. aureus*-derived products that promote or avert programmed cell death signaling in host cells. Finally, we highlight staphylococcal tactics for the manipulation of autophagy, a cell death-associated cytoplasmic degradation mechanism that sustains cellular homeostasis and survival.

## Apoptosis and Apoptotic Signaling Pathways

**Figure 2:**
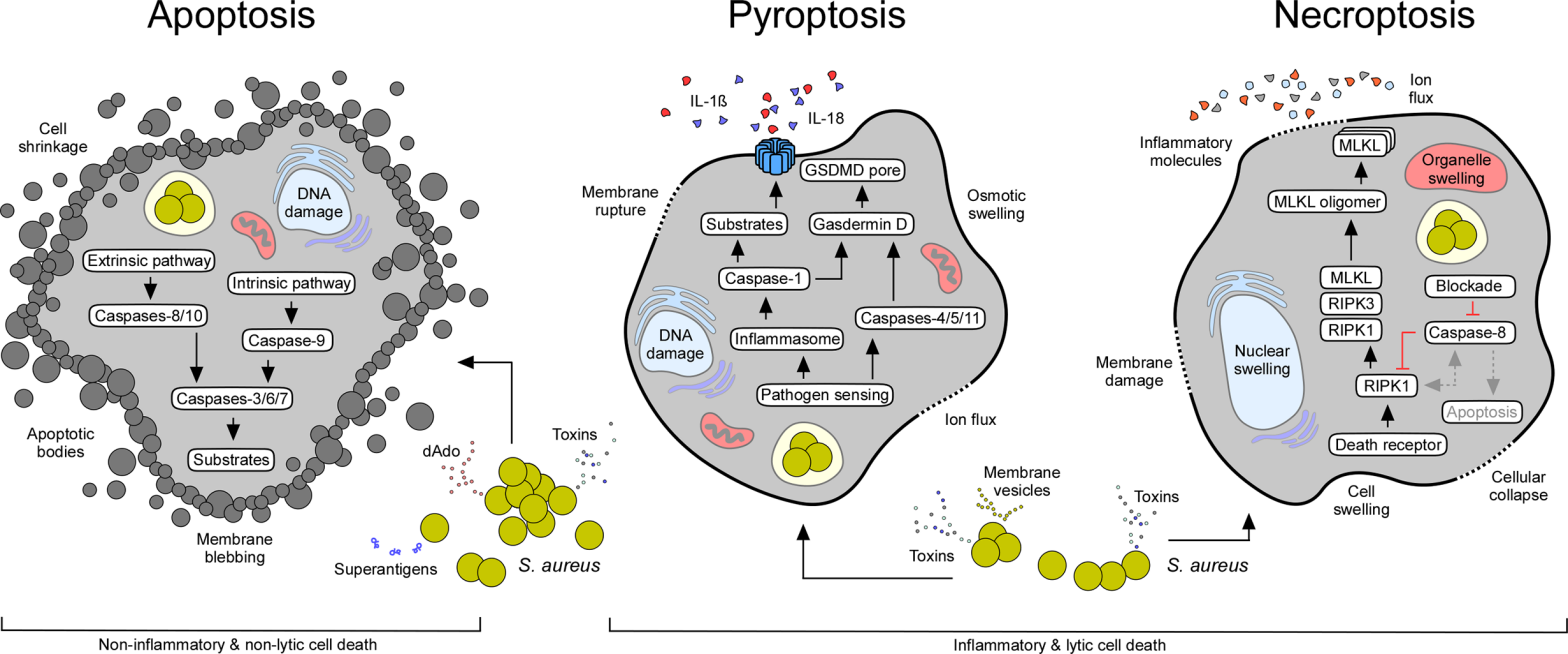
Staphylococcal interference with host cell death machineries. All major cell death modes including apoptosis, pyroptosis, and necroptosis may occur in response to extra- or intracellular staphylococci and their exoproducts (see **Table 1**). While apoptotic cell death is immunologically silent, pyroptosis and necroptosis cause strong inflammatory responses due to the release of pro-inflammatory molecules from injured host cells. Characteristic features and canonical signaling pathways of cell death modalities are indicated.

Apoptosis is an essential mechanism attributed to various physiological events. Apoptosis is considered an important component of multiple cellular processes and plays a significant role during normal development, organ shaping, homeostasis, and aging (19). Apoptosis is also favored by stress, lack of nutrition, and several other pathological conditions (19). Earlier work identified key genetic elements and two major signaling routes that regulate apoptosis in mammalian cells: the intrinsic (mitochondrial) and extrinsic (death receptor-mediated) pathways of apoptosis (**Figure 2**) (19). The extrinsic pathway is triggered by external signals and transmembrane death receptors (i.e., FasR or TNFR1) for activation of the death-inducing signaling complex (DISC) and initiator caspases-8 and -10; the intrinsic pathway is induced by internal stimuli, subcellular stress, and the release of apoptogenic proteins from injured mitochondria (**Figure 2**) (19). Microbial infections, DNA damage, cytotoxic stimuli, and various other pro-apoptotic signaling molecules promote permeabilization of the mitochondrial outer membrane, in a process mainly controlled by proteins of the Bcl-2 family (i.e., Bcl-2-associated X protein (Bax)) (20–22). Cellular stress favors oligomerization of Bax and Bak (Bcl-2-antagonist killer 1) and subsequent formation of pores in mitochondrial membranes (21, 23). Perforated mitochondria release cytochrome c and other pro-apoptotic proteins into the cytosolic space (19, 21, 24). Voltage-dependent anion-selective channels (VDAC) may enhance the release of mitochondrial pro-apoptotic factors by interacting with dedicated Bcl-2 family proteins (25–27). Cytosolic cytochrome c, together with dATP and the apoptotic protease activating factor 1 (APAF1), trigger the formation of the ultra-large apoptosome complex that activates the initiator caspase-9 (28, 29). Once caspase-9 (or caspases-8 or -10 in case of the extrinsic pathway of apoptosis) is activated, effectors caspases-3, -6, and -7 are proteolytically processed and converted to mature proteins that degrade defined target substrates; the ultimate result culminates with cell death exhibiting typical morphological features of apoptosis: membrane blebbing, cell shrinkage, DNA fragmentation, nuclear condensation, and formation of apoptotic bodies (**Figure 2**) (19).

## Apoptotic Cell Death in Response to *Staphylococcus aureus* Infections

Apoptotic cell death of hematopoietic and non-hematopoietic cells plays a significant role during *S. aureus* disease pathogenesis. During infection, *S. aureus* provokes apoptosis in a broad spectrum of target cells as a means to invade tissues, and to antagonize host immune defenses (18, 30). Depending on the type of tissue and staphylococcal isolate, apoptosis may occur *via* extrinsic-, intrinsic-, or caspase-2-mediated apoptotic signaling (31–36). *S. aureus* produces a vast array of pro-apoptotic virulence factors that predominantly encompass potent toxins and superantigens (**Table 1**) (17). Genetic variability amongst *S. aureus* isolates increases the repertoire of toxins and superantigens. All of these factors are secreted into the extracellular milieu and are endowed with membrane-damaging or toxigenic properties that interfere with apoptotic signaling cascades (17, 76). For example, the staphylococcal pore-forming toxins α-toxin, leukocidin AB (LukAB), or the Panton-Valentine-leukocidin (PVL), have been shown to prime apoptotic cell death in professional phagocytes and other cells (31–33, 36, 48, 49). Pore-forming toxin-mediated apoptosis involves potassium efflux from damaged cells and caspase-2-initiated cell death, or breakdown of the mitochondrial membrane potential, ultimately leading to the release of apoptogenic factors (e.g., cytochrome c) and activation of intrinsic death signaling pathway (31–33, 36). Staphylococcal superantigens (i.e., enterotoxin B) interact with T-cell receptors *via* major histocompatibility complex (MHC-II) molecules to stimulate biosynthesis and release of apoptogenic factors such as TNF-α, FasL, or IFN-γ (35, 44, 55). In this fashion, *S. aureus* triggers a pro-apoptotic milieu that induces extrinsic apoptosis in adjacent host target cells. Overall, toxin-mediated activation of apoptosis and subsequent killing of phagocytes eliminates primary host defenses essential for pathogen clearance. Pore-forming toxins are also thought to be key for the successful facultative intracellular lifestyle of *S. aureus* in non-professional phagocytes. Specifically, internalization of staphylococci by epithelial cells, endothelial cells, fibroblasts, keratinocytes, or osteoblasts can stimulate apoptotic cell death signaling (**Figure 2**) (40, 50, 77–82). In this manner, *S. aureus* not only escapes from host immune cell responses but also promotes tissue injury, and subsequent infiltration into deeper tissues, organs, or circulating body fluids. Following blood stream dissemination, *S. aureus* can successfully invade organ tissues to seed abscess lesions by initiating apoptotic death of surrounding cells in a manner independent of pore-forming toxins or superantigens (**Table 1**). *S. aureus* abscess formation involves two secreted enzymes, staphylococcal nuclease and adenosine synthase A (AdsA), that together convert neutrophil extracellular traps (NETs) into deoxadenosine (dAdo), a pro-apoptotic molecule, which kills phagocytes (37). dAdo-intoxication of macrophages involves uptake of dAdo by the human equilibrative nucleoside transporter 1 (hENT1), subsequent targeting of the mammalian purine salvage pathway, and signaling *via* dATP formation to activate caspase-3-dependent apoptosis and immune cell death (37–39). In this manner, macrophages are excluded from abscess lesions without causing inflammation thereby promoting the establishment of invasive disease (37, 39). More recently, Stelzner and colleagues discovered that intracellular *S. aureus* elaborates Staphopain A, a secreted cysteine protease, to trigger apoptosis in epithelial cells after translocation to the host cell cytosol (53). Staphopain B and the type-VII secretion system effector EsxA may also interfere with apoptotic cell death of human cells (**Table 1**) (47, 51, 52, 54). In summary, *S. aureus* exploits apoptosis to incapacitate macrophages and other host cells without provoking inflammatory responses; this facilitates infiltration of the bacteria in tissues and the establishment of persistent lesions filled with replicating staphylococci.

**Table 1:** Selected staphylococcal factors interfering with programmed cell death and autophagic signaling pathways.

Pathway	Staphylococcal factor	Category	Affected cells^1^	References
Apoptosis	AdsA-derived dAdo	Deoxyribonucleoside	Macrophages	(37–39)
	α-toxin	Pore-forming toxin	Epithelial cells, endothelial cells, T-cells, monocytes, eosinophils	(31, 33, 36, 40–42)
	Enterotoxin A	Superantigen	T-cells	(43)
	Enterotoxin B	Superantigen	Macrophages, T-cells, epithelial cells	(35, 44, 45)
	Enterotoxin H	Superantigen	Epithelial cells	(46)
	EsxA	WXG-like protein	Epithelial cells	(47)
	Leukocidin AB	Pore-forming toxin	Dendritic cells	(48)
	Panton-Valentine leukocidin	Pore-forming toxin	Neutrophils, macrophages, keratinocytes	(32, 49, 50)
	Peptidoglycan	Cell wall component	Platelets	(51)
	Protein A	Surface protein	Osteoblasts	(52)
	Staphopain A	Cysteine protease	Epithelial cells	(53)
	Staphopain B	Cysteine protease	Neutrophils, monocytes	(54)
	TSST-1	Superantigen	B-cells	(55)
Pyroptosis	α-toxin	Pore-forming toxin	Monocytes, macrophages, keratinocytes, microglial cells	(56–61)
	Extracellular vesicles	Membrane vesicles	Macrophages	(62)
	γ‐hemolysin	Pore-forming toxin	Microglial cells, macrophages	(61, 63)
	Leukocidin AB	Pore-forming toxin	Monocytes, dendritic cells	(48, 64)
	Panton-Valentine leukocidin	Pore-forming toxin	Monocytes, macrophages, neutrophils	(63, 65)
	Peptidoglycan^2^	Cell wall component	Macrophages	(66)
	Phenol-soluble modulins^3^	Cytolysin	Keratinocytes	(67)
Necroptosis	α-toxin	Pore-forming toxin	T-cells, macrophages	(68, 69)
	FumC	Fumarate hydratase	Keratinocytes	(70)
	Panton-Valentine leukocidin	Pore-forming toxin	Neutrophils	(32)
	Phenol-soluble modulins	Cytolysin	Neutrophils	(71)
Autophagy	α-toxin	Pore-forming toxin	Epithelial and epithelial-like cells (CHO), endothelial cells	(72–74)
	IsaB	Secreted and cell-surface-associated protein	Epithelial cells, macrophages	(75)

^1^For each individual factor and pathway listed, other target cells may exist. ^2^PBP2A-derived peptidoglycan; ^3^ caspase-1-independent mechanism.

## Pyroptosis and the Inflammasome

Unlike apoptosis, pyroptosis denotes a highly inflammatory state that largely depends on the activation of interleukin-1β (IL-1β)-converting enzyme also known as caspase-1 (**Figure 2**) (6). Caspase-1 is synthesized as an inactive zymogen in mammalian cells and was the first **c**ysteine-dependent **asp**artate-specific prote**ase** (caspase) discovered in scientific history (83). Processing and subsequent proteolytic cleavage of caspase-1 occurs within the inflammasome, a supramolecular complex that encompasses a member of NOD-like receptors (NLRs) (**Figure 2**) (6, 84). NLRs contain carboxy-terminal leucine rich repeats (LRR), a structural feature shared with Toll-like receptors (TLRs), which evolved to sense a large set of pathogen-associated danger signals, including bacterial or viral nucleic acids (85–88). In addition, inflammasome-associated NLRs are endowed with a variable N-terminal region that consists of a caspase activation and recruitment domain (CARD), or a pyrin (PYD) subunit that interacts with a CARD-domain containing adaptor protein (ASC) (CARD domains facilitate binding and proteolytic cleavage of caspase-1) (88–92). Following processing, catalytically active caspase-1 cleaves pro-forms of IL-1β, IL-18, and IL-33 into biologically active and secreted cytokines, ultimately leading to strong pro-inflammatory responses that dictate the recruitment of immune cells and pathophysiological outcome of disease (**Figure 2**) (6). Inflammasome activated caspase-1 also cleaves pro-Gasdermin D, the actual executor of pyroptosis (93, 94). Processing of Gasdermin D leads to the release of a plasma membrane pore-forming subunit (GSDMD-N domain) that interacts with acidic phospholipids found on the inner leaflet of mammalian plasma membranes (**Figure 2**) (95, 96). Together with distinct mechanisms such as microvesicle shedding (97), GSDMD-N-derived plasma membrane pores facilitate the rapid release of the aforementioned pro-inflammatory cytokines and intracellular molecules into the extracellular milieu, and ultimately drive swelling and osmotic lysis of host cells (**Figure 2**) (95, 96). Other caspases may also trigger pyroptosis (98–100). For example, caspases-4, -5, -11, as well as apoptosis executor caspase-3, can process pro-Gasdermins directly upon stimulation, thus impacting pyroptotic cell death and its characteristic morphological features (94, 98–100).

### *S. aureus*-Mediated Activation of Distinct Host Inflammasomes

*S. aureus* pathogenesis involves activation of distinct inflammasomes, a process that primarily depends on the infection site and staphylococcal stimulus involved. Pioneering work by Mariathasan et al. uncovered that exposure of NLRP3-deficient bone marrow-derived macrophages to replicating *S. aureus* drastically reduced the detectable amount of mature caspase-1, and secreted cytokines IL-1β and IL-18 (86). Subsequently, multiple other studies revealed that *S. aureus* pore-forming toxins contribute to this phenomenon, and trigger the formation of the NLRP3 inflammasome, cytokine release, pyroptosis, or pyroptotic-like cell death (**Table 1**). Purified α-toxin or α-toxin-containing *S. aureus* culture supernatants rapidly activate caspase-1 and NLRP3-dependent signaling in THP-1 cells or mouse macrophages (56, 57). Similarly, staphylococcal bi-component toxins LukAB or PVL induce processing of caspase-1 and release of pro-inflammatory cytokines by human phagocytes (64, 65). However, attempts to block the cognate host proteins with small molecule inhibitors only marginally suppresses α-toxin-, PVL-, or LukAB-mediated cell death (56, 64, 65). Even the genetic ablation of *CASP1* cannot prevent bacterial pore-forming toxin-dependent killing of host cells, demonstrating that distinct mechanisms or cross talk between different cell death modalities may contribute to toxin-induced cell death (56, 64). In agreement with this notion, a drop in intracellular potassium as a result of K^+^ efflux caused by pore-forming toxins or activation of the lysosomal cysteine protease cathepsin B provoke assembly of the NLRP3 machinery and cytokine release (64, 65, 101, 102). More recent work revealed that pyroptotic cell death is also driven by *S. aureus*-derived membrane vesicles (MVs) (**Table 1**) (62). MVs deliver lipoproteins and pore-forming toxins along with other pro-pyroptotic effector molecules to host cells thereby stimulating TLR2-mediated priming of the NLRP3 inflammasome, ultimately leading to gasdermin D-dependent release of pro-inflammatory cytokines and pyroptotic cell death (62). Combined with the canonical secretory pathway, this dual strategy of toxin-mediated destruction of innate immune cells secures *S. aureus* survival in hosts and establishment of invasive disease.

*S. aureus* can also target the NLRP3 inflammasome and pyroptotic signaling in a subset of non-immune host cells. Keratinocytes, when exposed to live *S. aureus*, culture supernatants, or staphylococcal toxins, produce elevated levels of IL-1β and IL-18, and exhibit pyroptotic characteristics (58, 67). *S. aureus*-induced skin inflammation and severity of dermal disease has been correlated with stimulation of the inflammasome and cytokine signaling (57, 58, 67, 103). Neither wild-type mice infected subcutaneously with a panel of toxin-deficient *S. aureus* mutants, nor *NLRP3*-, *ASC*-, or *CASP1*-deficient animals infected with wild-type *S. aureus* elicit NLPR3-dependent inflammatory responses and cytokine signaling (57). As a result, *ASC*^-/-^ or *IL-1β*^-/-^ mice fail to recruit neutrophils and other phagocytes to infectious foci, and develop significantly enlarged lesions in an experimental model of *S. aureus* skin infection (**Table 2**) (103). In line with these observations, impaired expression of *NLRP3*, *ASC*, and *CASP1* dampens neutrophil attraction in atopic dermatitis patients thereby increasing the risk of pathogen colonization and chronic skin inflammation (117). Yet, pyroptosis may also correlate with enhanced staphylococcal diseases, including traumatic osteomyelitis, central nervous system infections, and acute pneumonia (**Figure 1**) (59, 106, 118). As with skin infections, staphylococcal pulmonary disease and superinfections of lungs are associated with altered activity of the NLRP3 inflammasome (60, 107, 110). *S. aureus*-driven pneumonia induces additional inflammasome machineries such as NLRP6 (59). Of note, activation of the NLRP6 inflammasome during acute pneumonia negatively regulates pulmonary defenses, as *NLRP6*^-/-^ mice accelerate neutrophil recruitment and display increased resistance to staphylococcal lung infection (**Table 2**) (59). Lastly, the NLRP7 inflammasome senses intracellular staphylococci and acetylated lipoproteins, restricting bacterial replication and dissemination of disease **(**119**)**. However, the exact role of NLRP7 for *S. aureus* pathophysiology remains enigmatic. Collectively, *S. aureus* hijacks distinct inflammasomes and pyroptotic cell death modalities during infection, presumably to promote host invasion and immune evasion. Since *S. aureus*-induced activation of pyroptotic cell death elicits robust inflammatory and immune responses, pyroptosis may also contribute to host-mediated clearance of staphylococci.

**Table 2:** Selected cell death- and autophagy-associated host genetic determinants affecting *S. aureus* pathogenesis *in vivo*.

Pathway	Host factor^1^	Role during staphylococcal disease^2^	References
Apoptosis	*Bcl-2*	affects apoptosis in intestinal epithelial cells following pneumonia	(104)
	*Bid*	affects apoptosis in intestinal epithelial cells following pneumonia	(104)
	*CASP3*	suppresses macrophage infiltration into renal abscesses; affects staphylococcal clearance	(39)
	*CASP3/9*	promotes staphylococcal endophthalmitis	(105)
	*Fas-L*	impacts T-cell apoptosis in response to staphylococcal superantigens	(44)
	*PARP-1*	provokes staphylococcal endophthalmitis	(105)
Pyroptosis	*AIM2*	affects bacterial clearance in lungs of superinfected animals; protective role during CNS infection	(106, 107)
	*ASC*	protective role during CNS and skin infection; mediates increased mortality during influenza and bacterial superinfection; exacerbates outcome of pneumonia; controls of IL-1β and IL-18 production during skin infection	(57, 59, 103, 106, 107)
	*CASP1*	controls of IL-1β and IL-18 production during skin infection	(57)
	*CASP1/4*	promotes clearance of *S. aureus* from infected skin; enhances survival during sepsis	(108)
	*CASP1/11*	protective role during CNS infection	(106)
	*CASP11*	exacerbates lung infection	(109)
	*IL-1β*	protective function during skin infection	(103)
	*NLRP3*	controls of IL-1β and IL-18 production during skin infection; impairs lung infection; regulates bacterial burden during surgical wound infection	(57, 60, 107, 110, 111)
	*NLRP6*	exacerbates outcome of pneumonia	(59)
Necroptosis	*JNK*	detrimental effect during lung infection	(112)
	*MLKL*	protective role during dermal infection; enhances survival during sepsis; promotes chronic infections of the skin	(70, 108)
	*PPARα*	detrimental effect during superinfection	(113)
	*RIPK1*	protective function during dermal infection promotes chronic infections of the skin	(70, 108)
	*RIPK3*	provokes skin infection; promotes superinfection	(108, 113)
Autophagy	*ATG16L1*	enhances survival during bloodstream infection; protective role during lung infection; contributes to biogenesis of α-toxin-neutralizing exosomes	(73, 114)
	*LC3*	protective role during bloodstream infection and pneumonia	(73)
	*SQSTM1*	protective function during *S. aureus* infection (zebrafish larvae)	(115, 116)

^1^Gene names are indicated. ^2^Analyzed by using (conditional) knock-out or inhibitor-treated mice in comparison to wild type or control animals.

## Necroptotic Cell Death and Its Pathological Features

Necrosis stems from the Greek word “nekros” (dead body) and represents a passive and uncontrolled form of cell death. While initially considered to represent an accidental form of cell death that lacks a defined signaling network, recent work uncovered the existence of multiple pathways contributing to the control of necrosis (120). The prototypical form of regulated necrosis, necroptosis, requires several kinases, including the mixed lineage kinase domain-like protein (MLKL) and receptor-interacting protein kinases 1 and 3 (RIPK1, RIPK3); regulated necrosis also requires dedicated plasma membrane receptors and their ligands (**Figure 2**) (121–124). More precisely, necroptotic signaling largely depends on death receptor mediated signaling molecules (i.e., Fas or TNF) that interfere with their cognate plasma membrane receptors, leading to the formation of a stable, but short-lived RIPK1- and TRADD (TNFR1-associated death domain)-dependent receptor-bound complex I (122, 125–127). In addition to RIPK1 and TRADD, this multimeric complex encompasses cellular inhibitor of apoptosis proteins 1 and 2 (cIAP1/cIAP2), TNF receptor-associated factor 2 (TRAF2) and TRAF5. Together, TRAF2 and TRAF5 mediate polyubiquitination of RIPK1 (126, 128–130). Ubiquitination of RIPK1 features the assembly of the inhibitor of nuclear factor-κB (NF-κB) kinase (IKK) complex, which promotes the upregulation of NF-κB pathway and several anti-apoptotic genes, including the FLICE-like inhibitory protein (FLIP) (120). However, deubiquitination of RIPK1 *via* cylindromatosis (CYLD) and other deubiquitinases destabilizes complex I, a crucial step that promotes interaction of RIPK1 with FADD (FAS-associated death domain), TRADD, RIPK3, pro-caspase-8, and the long isoform of FLIP (FLIP_L_) to form the TRADD-dependent complex II (126, 131–134). Subsequently, pro-caspase-8 and FLIP_L_ form a heterodimer complex that cleaves and inactivates RIPK1, RIPK3, and CYLD to prevent necroptosis (135–139). Pro-caspase-8 homodimerization induces auto-proteolysis and formation of active caspase-8 that processes the apoptosis-executing caspases 3 and 7, ultimately promoting apoptotic cell death (120). Nevertheless, chemical or pathogen-induced blockade of caspase-8 provokes the complexation and autophosphorylation of RIPK1 and RIPK3 that leads to the assembly of an intracellular machinery designated necrosome (**Figure 2**) (140). Upon necrosome formation, downstream signaling leads to the recruitment of MLKL, a pseudokinase that interacts with the inner leaflet of plasma membranes in its phosphorylated state (123, 141–143). In this manner, MLKL disrupts the integrity of the cell thereby promoting necroptosis (**Figure 2**). Apart from death-receptor-mediated necroptosis, regulated necrosis can further be triggered by TLR-mediated signaling or certain intracellular stimuli that lead to the formation of non-classical necrosomes (144, 145). Moreover, DNA damage can activate RIPK3 and biogenesis of another necroptosis-executing multiprotein complex termed ripoptosome (146). Overall, necroptosis constitutes a caspase-independent form of programmed cell death that is morphologically characterized by massive organelle and cellular swelling, and rupture of plasma membranes (**Figure 2**). Hence, regulated necrosis causes robust inflammatory responses and severe tissue injury, thus affecting the pathophysiology of many infectious and non-infectious diseases.

## Exploitation of Necroptotic Signaling by *Staphylococcus aureus*

The discovery of necroptotic signaling cascades enabled the staphylococcal research community to uncover the significance of necroptosis-dependent cell death in the pathophysiology of *S. aureus* diseases. Initial work aimed to identify microbial and host determinants that modulate necroptotic cell death during acute and persistent infections, specifically in the context of staphylococcal pulmonary disease (**Tables 1** and **2**). As expected, staphylococcal pore-forming toxins including α-toxin promote tissue damage and necroptotic cell death in immune and epithelial cells during lung infection (68, 147). Moreover, *S. aureus* phenol-soluble modulins (PSM peptides) constitute potent catalysts of necroptosis as these cytolytic peptides activate necroptotic death of host phagocytes *via* induction of MLKL phosphorylation, ultimately leading to exacerbated outcomes of staphylococcal pulmonary infections (71). Although most of these toxins have distinct receptors, all variants exhibit potent immunomodulatory properties that together trigger assembly of the necrosome, and subsequent necroptotic cell death (68, 148, 149). However, some of these studies revealed that *S. aureus* toxin-mediated necroptosis may directly interfere with pyroptotic signaling pathways. For example, it was found that the pharmacological inhibition of MLKL dampens caspase-1 activation and pyroptotic signaling in host cells upon staphylococcal stimulation (68, 150). Thus, it is not surprising that mice lacking the NLRP6 inflammasome exhibit both, reduced pyroptotic and necroptotic signaling following pathogen challenge (59). Nevertheless, pharmacological and genetic perturbation of key modulators of necroptosis such as MLKL, RIPK1, or RIPK3 can clearly protect human and murine macrophages as well as neutrophils from CA-MRSA strain USA300 and its secreted toxins (68, 148–151). In line with these findings, *RIPK3* knock-out mice display increased resistance during experimental *S. aureus* lung infection, an effect attributed to anti-inflammatory CD206^+^ and CD200R^+^ alveolar macrophages that accumulate in lungs and may accelerate the clearance of staphylococci (**Table 2**) (68). Also, *in vivo* blockade of c-Jun N-terminal kinases (JNK1 and JNK2), both of which are known to trigger TNF- and TLR-induced necroptotic cell death, or genetic ablation of the peroxisome proliferator-activated receptor α (PPARα), a ligand-activated transcription factor and suppressor of NF-κB activation, rescued mice from fatal staphylococcal lung disease, even under conditions that mimic bacterial superinfections (**Table 2**) (112, 113). Zhou et al. exploited RNAIII-inhibiting peptide as an anti-virulence therapeutic approach to prevent PSM- and necroptosis-dependent lung injury in mice nasally infected with CA-MRSA strain USA300 (71). RNAIII is the effector of accessory gene regulator (Agr) and RNA-III inhibiting peptide blocks *S. aureus* quorum sensing and biogenesis of PSMs toxins during acute lung infection (71). Together, this compelling work underscores the importance and clinical relevance of necroptosis during *S. aureus* pulmonary disease, and suggests that staphylococcal exoproducts may simultaneously trigger distinct and genetically conserved cell death programs in mammalian cells to establish infection (**Figure 2**).

*S. aureus* can also trigger necroptosis in the absence of pore-forming toxins (**Table 1**). Wild-type *S. aureus* or its *hemB* variant stimulate host cell glycolytic activity and formation of mitochondrial reactive oxygen species in skin cells in a manner that promotes necroptotic cell death without the contribution of bacterial toxigenic molecules (70). Mutations in the *hemB* gene and other metabolic genes arise spontaneously during infection and are identified on laboratory medium as small colony variants (SCVs). SCVs represent auxotrophic subpopulations, which while less virulent, are able to persist within host cells and are associated with chronic infection (152). Intracellular *hemB* variants induce the biogenesis of bacterial fumarate hydratase that degrades cellular fumarate, a known inhibitor of the glycolytic pathway of mammalian cells (70). In this manner, SCVs activate necroptosis to promote persistence in skin cells (70). Induction of necroptosis may also represent a selective response of host keratinocytes to eradicate the invading pathogen as mice lacking key elements of the necroptotic signaling pathway such as *MLKL* exhibit significantly enlarged wounds and higher bacterial loads during *S. aureus* experimental skin infection (**Table 2**) (108). Although these animals recruited more immune cells to the primary skin lesion and produced elevated levels of pro-inflammatory cytokines due to excessive activation of caspase-1, they failed to clear replicating staphylococci (108). Consistent with these findings, MLKL-proficient animals display enhanced survival rates over time in a *S. aureus* murine bacteremia model, further suggesting that induction of necroptosis may be beneficial for the host (108). Collectively, these observations suggest that necroptosis may restrict hyper-inflammatory immune responses during skin or blood stream infections thereby serving as a protective mechanism that promotes bacterial eradication from infected hosts (108). However, *S. aureus* may also exploit the necroptotic signaling pathway to combat resident and recruited innate immune cells during acute or chronic infections.

## Autophagy and Autophagic Cell Death

**Figure 3:**
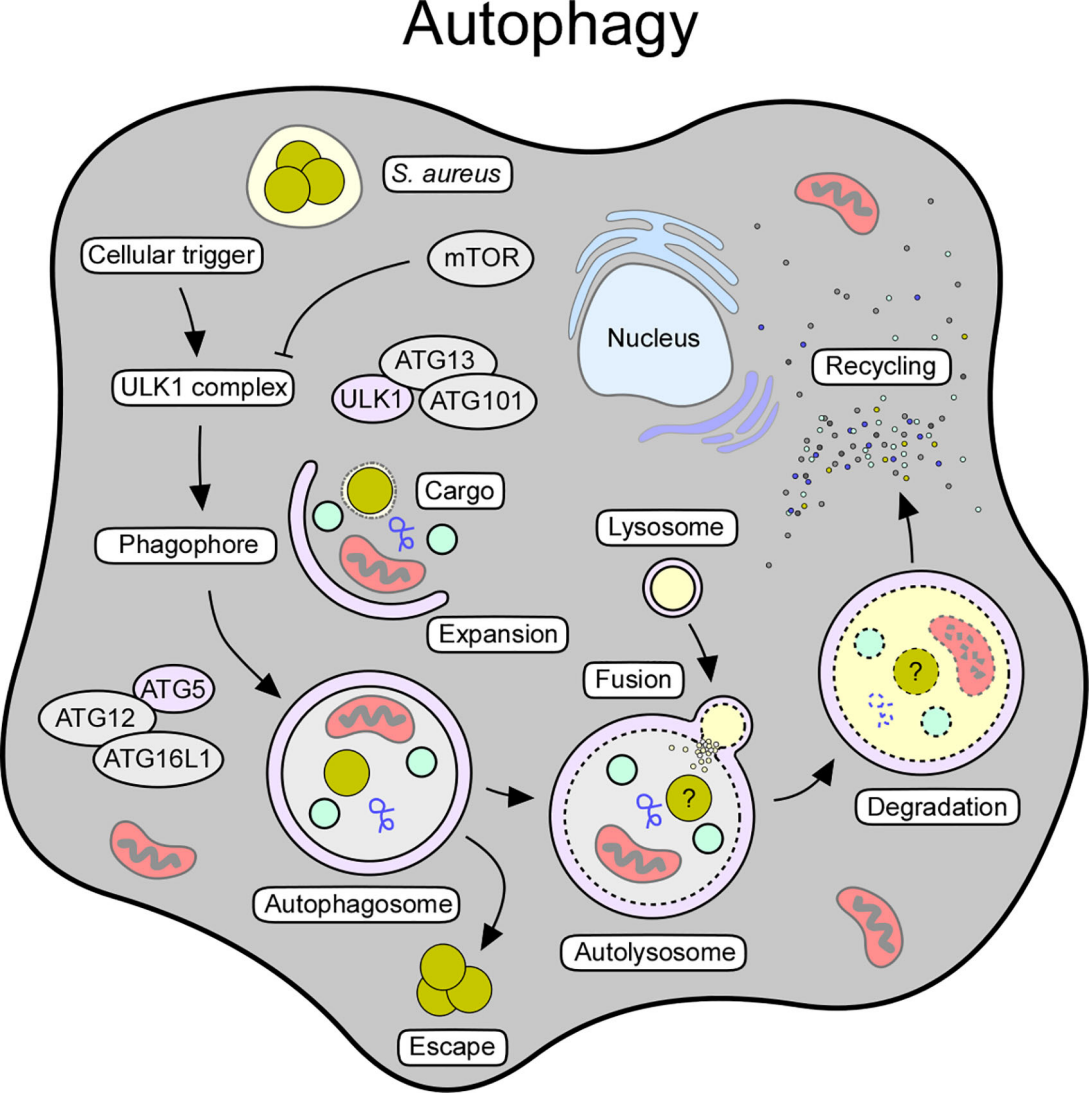
Cellular and molecular features of autophagy. Autophagy can be triggered by various stimuli, including starvation, oxidative stress, pathogens, or pathogen-derived products. The autophagic signaling process can also be initiated in response to intracellular staphylococci and their secreted toxins. Release of toxins and other virulence determinants presumably prevents maturation of autophagosomes thereby boosting staphylococcal escape from these structures. Key factors and crucial steps of the autophagy pathway are highlighted.

Autophagy (from Greek, “self-eating”) constitutes a highly conserved process that controls cellular development, homeostasis and survival (153). This housekeeping mechanism represents a protective platform and cellular recycling system that overcomes several pathological states or harmful conditions (153, 154). Comprehensive work uncovered mechanistic details and the existence of at least three forms of autophagy (micro- or macroautophagy, and chaperon-mediated autophagy), all of which rely on lysosome-based degradation of unnecessary or detrimental molecules (154–156). Herein, we focus on macroautophagy, which resembles the canonical form of autophagy and main mechanism known to interfere with staphylococcal infections (**Figure 3**).

Macroautophagy (referred to as autophagy) requires a cellular trigger (i.e., starvation or oxidative stress) that abrogates mTOR-mediated suppression of autophagic signaling, thus leading to the assembly of the autophagy initiator complex (Unc-51-like kinase 1 [ULK1] complex) (153, 157) (**Figure 3**). This multimeric protein complex consists of ULK1, autophagy-related proteins (ATG)-13 and ATG101, and FIP200, a focal adhesion kinase family-interacting protein (153, 158). Following priming, the ULK1 complex phosphorylates AMBRA (activating molecule in Beclin-1-regulated autophagy protein 1) as part of the ATG14-, VPS15-, VPS34-, Beclin-1-, and p115-consisting PI3KC3 complex I (153, 159). Together with the ULK1 complex, this autophagy-modulating element initiates the biogenesis of phagophores, and subsequently promotes the generation of phosphatidylinositol-3-phosphate (PI3P), which serves as a docking scaffold for WD repeat domain phosphoinositide-interacting proteins (WIPIs) and other effector molecules (153, 154). Phagophore-associated WIPIs in turn recruit an array of ATG proteins (that is ATG16L1 and the ATG5-ATG12-ATG3 conjugate), which facilitate the ATG3-driven conjugation of ATG class 8 proteins such as LC3 (microtubule-associated protein light chain 3) to phosphatidylethanolamine (PE) (153, 160). In this manner, conjugated LC3 is lipidated and readily incorporated into autophagic membranes (153, 154). Membrane-associated LC3/ATG8 conjugates capture and recruit labeled (unwanted) molecules *via* selective autophagy receptors such as sequestosome 1 (SQSTM1/p62) (153, 154). Together with ATG9-positive vesicles and cellular membrane material, ATG8s further promote phagophore expansion and sealing around selected cargo, ultimately leading to the formation of the autophagosome (153, 154) (**Figure 3**). Once autophagosomes are fully assembled, ATG family proteins disassociate to enable maturation and fusion of the autophagosome with acidic hydrolases-containing lysosomes (153, 154) (**Figure 3**). In this manner, autolysosomes recycle cellular trash, intracellular pathogens, or damaged organelles into elementary building blocks required for macromolecule biosynthesis or energy supply (154). Thus, autophagy represents a major cytoprotective mechanism that sequesters cytoplasmic material in double-membraned vesicles for subsequent detoxification and degradation. Excessive induction of autophagy can also trigger autophagic cell death (ACD), as autophagy and other cell death signaling pathways are interconnected and together influence the fate of dying cells (161).

## Staphylococcal Interference With Autophagic Signaling

Many intracellular pathogens such as *Mycobacterium tuberculosis* have developed refined strategies to antagonize autophagic signaling pathways during infection (162, 163). Owing to its ability to replicate in professional- and non-professional phagocytes, *S. aureus* is considered to be a facultative intracellular pathogen. This raises the possibility that *S. aureus* may suppress autophagy to cause persistent infection and chronic disease. Accumulating evidence suggests that *S. aureus* is indeed able to subvert autophagic responses (72, 164). Initial studies by Schnaith et al. demonstrated that *S. aureus* rapidly transits from endosophagosomes to LC3-positive autophagosomes upon invasion of HeLa cells (164). More recent work revealed that recruitment of *S. aureus* to phagophores requires SQSTM1/p62 and other autophagy receptor proteins that enable efficient tagging of staphylococci by neutrophils, fibroblasts, or keratinocytes (115, 116, 165). Although these investigations suggest that host cells may use autophagosomes to encircle intracellular staphylococci, *S. aureus*-containing autophagosomes cannot fuse with lysosomes and thereby fail to clear intracellular staphylococci (164). Autophagic vesicles and non-acidified phagosomes rather constitute a survival containment for host cell-engulfed staphylococci; *S. aureus* is able to exit these vesicles by secreting autophagosome-damaging toxins and other virulence determinants (116, 165, 166) (**Figure 3**). Indeed, α-toxin-proficient *S. aureus* or purified α-Hemolysin promote the initiation of an autophagic response but prevent maturation of autophagosomes (**Table 1**) (72). Concomitantly, autophagosome-associated staphylococci block autophagosome maturation by initiating the phosphorylation of the mitogen-activated protein kinase MAPK14 (p38α) (MAPK14/p38α) (165). Upon phosphorylation, activated MAPK14 traffics to autophagosomes where it inhibits autophagosome maturation and fusion with acidified lysosomes (167). *S. aureus* also deploys the immuno-dominant surface antigen B (IsaB), a secreted and cell surface-associated protein, to limit the autophagic flux in host cells thereby enhancing intra-host cell survival (**Table 1**) (75). Since *isaB* expression levels correlate with improved host colonization, IsaB-mediated subversion of autophagy may also promote host-to-host transmission of highly transmissible MRSA isolates (75). Exploitation of autophagic responses has further been observed in dendritic cells, where staphylococci accumulate in autophagosomes in an Agr-dependent manner as well as in keratinocytes or bovine mammary epithelial cells (168–170). *S. aureus*-mediated manipulation of the central carbon metabolism of host cells, as recently described for HeLa cells exposed to CA-MRSA strain USA300, promotes autophagic signaling and intracellular proliferation of bacteria (171). Specifically, NMR- and MS-based profiling of MRSA-infected cells revealed the conspicuous metabolic starvation of infected host cells, a typical trigger of autophagy sensed by the autophagy master regulator mTOR (171). Autophagy also contributes to innate immune cell defenses during staphylococcal disease pathogenesis, particularly in the context of infection control and tolerance to bacterial toxins (**Table 2**). Recent work by Gibson and colleagues suggested that autophagy governs cytosolic surveillance of replicating staphylococci in neutrophils (115). Using a zebra fish infection model, the investigators demonstrated that SQSTM1/p62 along with LC3 targets neutrophil-engulfed staphylococci for subsequent degradation *in vivo*, thus illustrating the protective potential of autophagy during staphylococcal infections (115). In agreement with this study, Maurer et al. discovered that autophagy diminishes host susceptibility to acute *S. aureus* infections, as autophagy-deficient mice (here: *ATG16L1*-hypomorph [*ATG16L1*^HM^] or *LC3*^-/-^ mice) display hypersensitivity towards *S. aureus* (73). Remarkably, increased mortality of *ATG16L1*^HM^ mice during both, sepsis or acute pneumonia, correlated with the biogenesis of staphylococcal α-toxin and its endothelial-damaging properties, and with elevated protein levels of ADAM10, the α-toxin receptor (73). Subsequent work by the same group uncovered that TLR9-sensed bacterial and CpG DNA along with ATG16L1 and other ATG proteins promote the release of ADAM10-containing exosomes during infection (114). These secreted exosomes capture and neutralize α-toxin and other bacterial toxins, a striking feature that protects the host from toxinosis and severe clinical syndromes (114). Together, these studies uncovered a crucial role of autophagy during staphylococcal infections. While *S. aureus* is able to hijack autophagosomes to elude from phagocytic killing and innate immune cell defenses, autophagy contributes an important host defense mechanism for the elimination of MRSA and other bacterial pathogens.

## Concluding Remarks

*S. aureus* provokes strong host responses during infection but circumvents the host’s immune system by secreting an extraordinary repertoire of virulence factors. Together these factors help subvert the complement system, the activity of immune cells (phagocytosis, chemotaxis, NETs formation) or promote their killing (76, 172, 173). The selective exploitation of host cell death machineries constitutes an additional strategy that secures invasion, spread, and intra-host survival of this bacterium. *S. aureus*-mediated demolition of host tissues and immune cells involves all key mechanisms by which programmed host cell death can occur, including immunologically silent apoptosis and highly inflammatory signaling pathways such as pyroptosis. Several outstanding questions remain to be examined. Does *S. aureus* gain any advantage by provoking both non- and pro-inflammatory cell death programs? This answer may depend on the environment where the pathogen proliferates as distinct host defense arsenals may be triggered in different organ tissues. For example, deep-seated abscess formation is accompanied by the biosynthesis of apoptogenic dAdo from NETs, allowing *S. aureus* to selectively kill macrophages through apoptosis (37, 39). In this environment, *S. aureus* converts host molecules to both toxigenic and immuno-suppressive products and the infected organ fails to alert the immune system of the presence of bacteria (37, 39). On the contrary, abscess lesions in the skin elicit necroptosis, toxin-mediated activation of the NLRP3 inflammasome, and a massive recruitment of neutrophils that release pro-inflammatory cytokines such as IL-1β (103, 108, 174). Since genetically modified mice with lesions in the pyroptotic or necroptotic signaling pathway develop larger skin lesions and exhibit impaired disease outcome during bacteremia models, it appears reasonable to assume that certain cell death modes may selectively be activated by the host to limit the severity of staphylococcal infections (103, 108). In line with this model, the cell death-driven magnitude of inflammation determines the outcome of *S. aureus* disease and local pathology, further demonstrating that pro-inflammatory death cascades may be in favor of the mammalian host (108). If so, one wonders why *S. aureus* is unable to subvert pro-inflammatory host cell death modes through anti-pyroptosis or anti-necroptosis mechanisms. Presumably, the extraordinary life cycle of *S. aureus* requires a delicate balance between immunologically silent and inflammatory death signaling pathways in order to develop disease. Alternatively, inflammatory death signaling cascades may promote dissemination during infection or transmission to other hosts. Indeed, excessive inflammation during skin and systemic diseases is generally believed to correlate with exacerbated disease outcomes and increased mortality rates, and may therefore represent a selective infection strategy by *S. aureus* to establish infection (66, 108). Concomitantly, coordinated and precise perturbation of different cell death programs and cytoprotective autophagic signaling routes may help the pathogen shift from an invasive to a persistent lifestyle, thereby contributing to its global success in both healthcare facilities and the community.

Overall, *S. aureus*-mediated manipulation of major cell death programs, autophagy, and contributing signaling pathways substantially affects staphylococcal disease pathogenesis and clinical manifestations in many aspects. Unravelling all facets and principle mechanisms by which *S. aureus* modulates host cell death, along with the identification of contributing host genetic determinants, may aid the design of new therapeutic approaches to combat MRSA and other drug-resistant bacterial pathogens that exploit host cell death machineries during acute or chronic infections.

## Author Contributions

VW performed the literature review and data collection, and prepared the manuscript draft and figures. DM provided revisions and comments. All authors contributed to the article and approved the submitted version.

## Conflict of Interest

The authors declare that the research was conducted in the absence of any commercial or financial relationships that could be construed as a potential conflict of interest.
